# Crossover trial of an audience response system application for smartphone in undergraduate medical students

**DOI:** 10.15694/mep.2018.0000215.1

**Published:** 2018-09-18

**Authors:** Camille Lours, Pierre Sujobert

**Affiliations:** 1 Université Lyon 1-Faculté de médecine Lyon Sud-Hospices Civils de Lyon

**Keywords:** Audience response system application, smartphone, crossover trial

## Abstract

This article was migrated. The article was marked as recommended.

**Objectives:** Audience response systems (ARS) using dedicated devices have been shown to enhance interactivity, leading to an increase in knowledge acquisition. ARS applications for smartphones are easier to use, but the benefits of these applications might be negated by deleterious effects of smartphone usage on concentration. We investigated whether an ARS smartphone application (Socrative ^TM^) is feasible and increases student satisfaction and knowledge acquisition.

**Methods:** We performed a crossover study in the setting of a hematology course for second-year medical students. Two hundred and forty nine students were included in the study and analyzed for their access to the ARS application through a smartphone. The same interactive lectures were proposed by the same teachers. The first group (n=119) was asked to answer questions with the ARS application during the first 3 lectures, and without the application during the last 3 lectures; and conversely for the second group (n=130). The analysis of the final results was restricted to 146 students having attended to at least 5 of the 6 lectures and having a smartphone enabling the use of the ARS application. Student opinion was measured through questions based on a Likert scale, and knowledge acquisition was measured at short and long-term through multiple choice questions assessing either the first three lectures or the last three lectures.

**Results:** Most of the students (86%) had a smartphone enabling the use of the application. They were satisfied by the use of the application (93%), and found that it increased both interactivity (92%) and concentration (68%). There was no difference in knowledge scores at short or long term.

**Conclusion:** The use of an ARS application for smartphone is feasible and increases the satisfaction of the students, their concentration and the interactivity of the lectures. However, this does not translate into a measurable increase in knowledge acquisition.

## Introduction

The use of audience response systems (ARS) provides teachers immediate feedback about the level of understanding of the students in the classroom, thus enabling them to offer alternative explanations of the concepts that have been insufficiently understood. They have been shown to increase the level of attention of the students, by moving from didactic to interactive teaching with peer-to-peer discussions ( Bergtrom, 2006; Caldwell, 2007). Consequently, ARS have the potential to increase the learning performance, even if their effect on knowledge retention seems at best marginal ( Hunsu, Adesope and Bayly, 2016). As underlined in a meta-analysis, the potential benefit of ARS is highly variable, and depends on the teaching context; evaluation of these devices should therefore be conducted under conditions as close as possible to real-life use, including education level, class size, and discipline. ^4^ In medical education, ARS have been shown to slightly improve knowledge scores (+4.5%) in a meta-analysis of 21 controlled trials from the Best Evidence Medical Education collaboration group ( Nelson *et al.*, 2012). However, most of the studies compared interactive lectures with ARS to traditional non-interactive lectures, thus demonstrating the value of question-based pedagogy instead of demonstrating the value of ARS itself. As a consequence, Nelson et al. underlined that controlled studies are warranted to better assess the potential of ARS to improve medical education.

More recently, various ARS applications have been developed for smartphones. They are easier to use because they do not require prior preparation of equipment ( Gooi *et al.*, 2014), and some of them are freely available. One potential limitation of these applications is the level of equipment among the students. More importantly, concerns have been expressed regarding the use of smartphones in the context of a lecture, especially because of the risk of being distracted by the use of other applications (such as social media or messaging) ( Siddiqui, Iqbal and Azizi, 2017). Indeed, smartphone usage has been associated with impaired knowledge acquisition among first year faculty students ( Baert *et al.*, 2018).

In order to assess the potential of ARS applications for smartphone in the context of undergraduate medical education, we conducted a crossover trial assessing both non-cognitive and cognitive effects of an ARS application in undergraduate hematology teaching.

## Methods

### Setting

The study was proposed to the 254 second-year medical students attending the hematology course of the medicine faculty Lyon Sud Charles Mérieux, Université Lyon 1, Lyon, France. At the end of this 12-hour course, the students are expected to be able to explain the basic physiology of hematopoiesis, to describe the principles of the nosological classification of hematological malignancies, to analyze normal and pathological blood cell count in order to choose appropriate additional testing, and to formulate the adequate etiological hypothesis for the main hematological syndromes.

**Figure 1.**
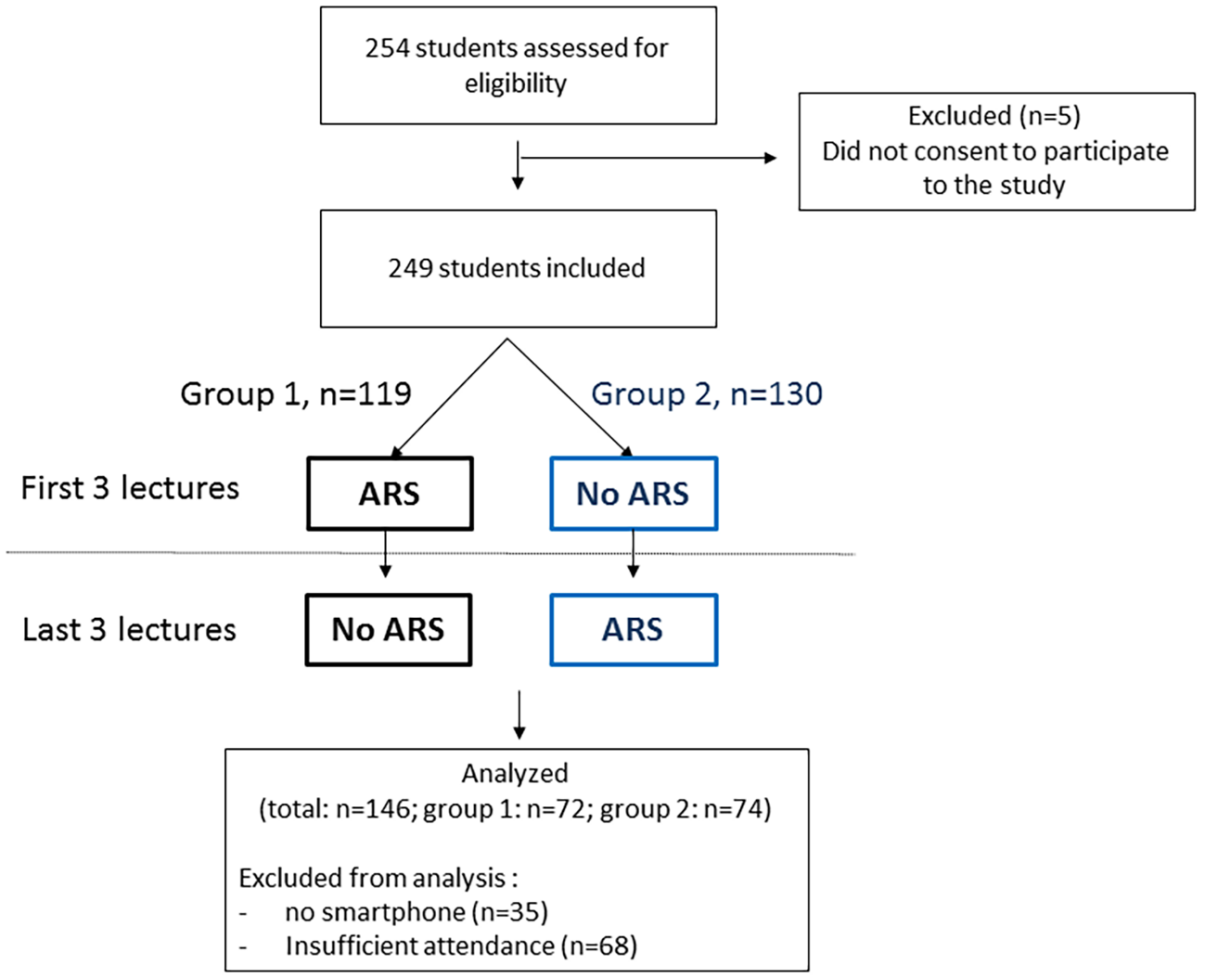
CONSORT diagram showing the flow of participants through each stage of crossover trial.

Two hundred and forty nine students having provided their consent were included in the study and analyzed for the access to the ARS application on a smartphone. The students were divided in two groups (group 1, n=119; group 2, n=130). Six interactive 2-hour lectures were given between September and December 2017 to both groups, with exactly the same questions testing the understanding of the lecture approximately every 20 minutes. For group 1, the ARS application was used during the first three lectures only (part 1), and for group 2, the ARS application was used for the three last lectures only (part 2; Figure 1). For the study the Socrative ^TM^ application was chosen ( https://www.socrative.com), because it is freely available and easy to install on iOS or android platforms.

The analysis of the effects of the ARS application on student’s satisfaction and knowledge acquisition was restricted to the 146 students possessing a smartphone that enables to use the Socrative ^TM^ application and having attended to at least 5 of the 6 lectures.

### Outcomes

The opinion of the students was measured through four anonymous questions added to the end of the multiple choice question-based exam for the hematology module. The students were asked about their overall satisfaction regarding the use of the ARS application, the consequence of the ARS application use on their concentration, on lecture interactivity, and on the potential waste of time induced by the use of the ARS application. We used a standardized Likert scale to quantify the responses. The Chi-square test was used to assess if the distribution of the responses was significantly different from a random distribution.

Short-term knowledge acquisition was evaluated through the module exam (20 multiple choice questions) two weeks after the last lecture. Half of the questions tested knowledge explained during the first 3 lectures (part 1) and half tested knowledge from the 3 last lectures (part 2). Three questions for each part were exactly the same as those used during the lectures; long-term knowledge retention was evaluated using these questions 3 months after the last lecture at the time of an exam for another module. The students were not informed about this evaluation, which had no impact on their exam results. A Mann-Withney test was used to assess the statistical significance of the mean result of the two groups in each part of the program.

## Results/Analysis

Among the 254 students, 249 provided their consent to be included in the study. There were 35/249 (14%) students who did not have a smartphone enabling access to the ARS application and were thus excluded from the analysis of the effects of the ARS application on student satisfaction or knowledge acquisition. Sixty eight other students were excluded from the analysis because they attended less than 5 of the 6 lectures. A total of 146 students were analyzed (72 in group 1, 74 in group 2; Figure 1).

There was no significant difference between the 2 groups regarding demographic variables (age, sex) and the results obtained in the first year exam ( Table 1).

**Table 1.** Comparison of the two group’s baseline characteristics.

	group 1	group 2	p value
**sex ratio** (H/F)	0,29 (35/119)	0,32 (42/130)	0,681
**age** (mean +/- sd, years)	20 +/- 2,1	20,3+/- 3,1	0,356
**first year exam result** (mean rank/235)	131	126	0,8054

### Student opinion

**Figure 2.**
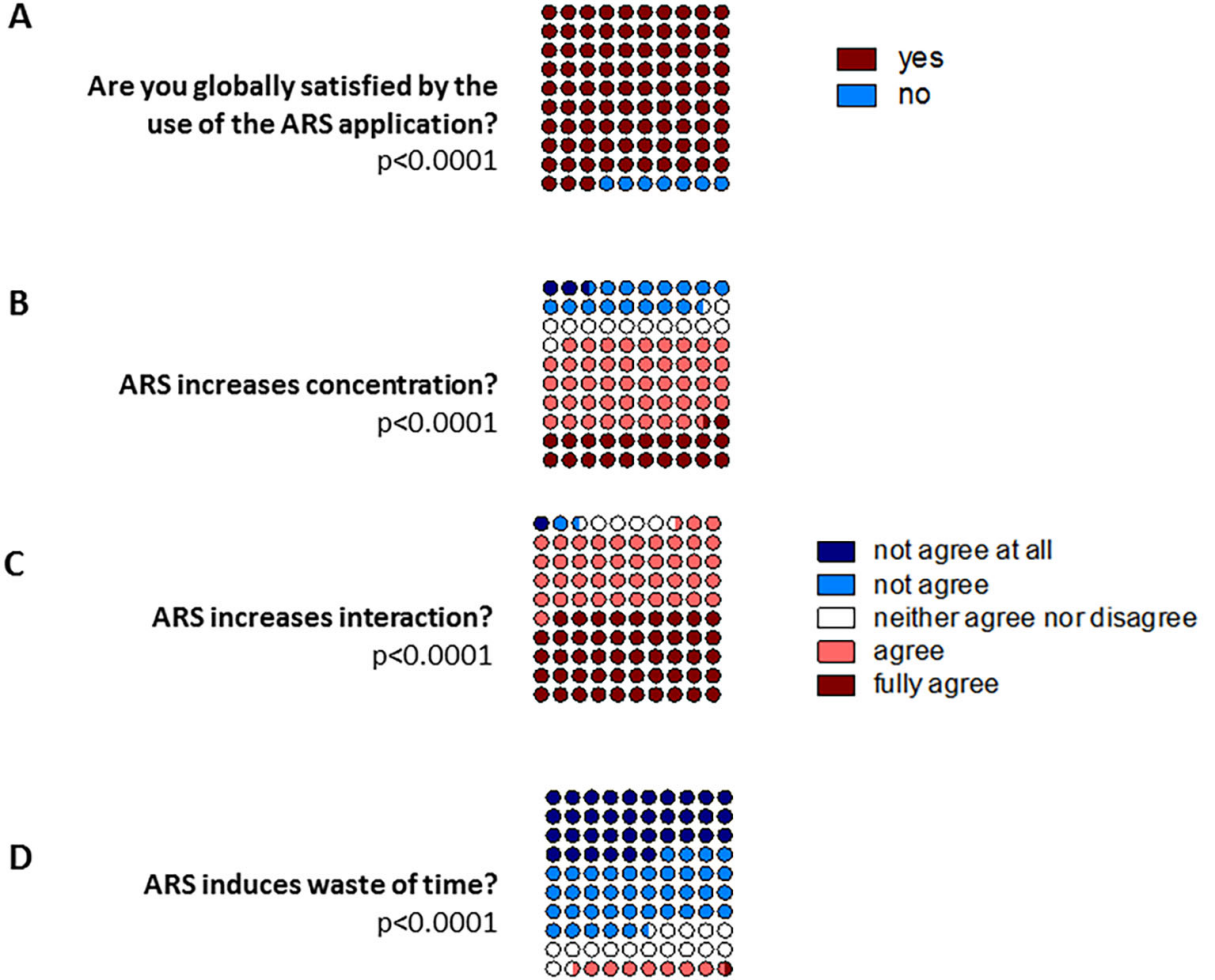
Evaluation of the satisfaction of the students

Most of the students (93%) were satisfied with the use of the ARS application ( Figure 2A). More precisely, 68% agreed or fully agreed with the proposition that the application increases their concentration during the lecture ( Figure 2B), and 92% agreed or fully agreed with the proposition that the ARS application increases the interactivity of the lecture, as compared with the same lecture without the application ( Figure 2C). Most of the students (75%) did not agree or did not agree at all with the proposition that the application is responsible for a waste of time during the lecture ( Figure 2D).

During the faculty exam, the students were asked to evaluate their global satisfaction about the use of the audience response system (ARS) application **(A)**, and to what extent they agree with the fact that the ARS increases concentration **(B)**, interaction **(C)** or induces time loss **(D)**.

### Knowledge evaluation

**Figure 3.**
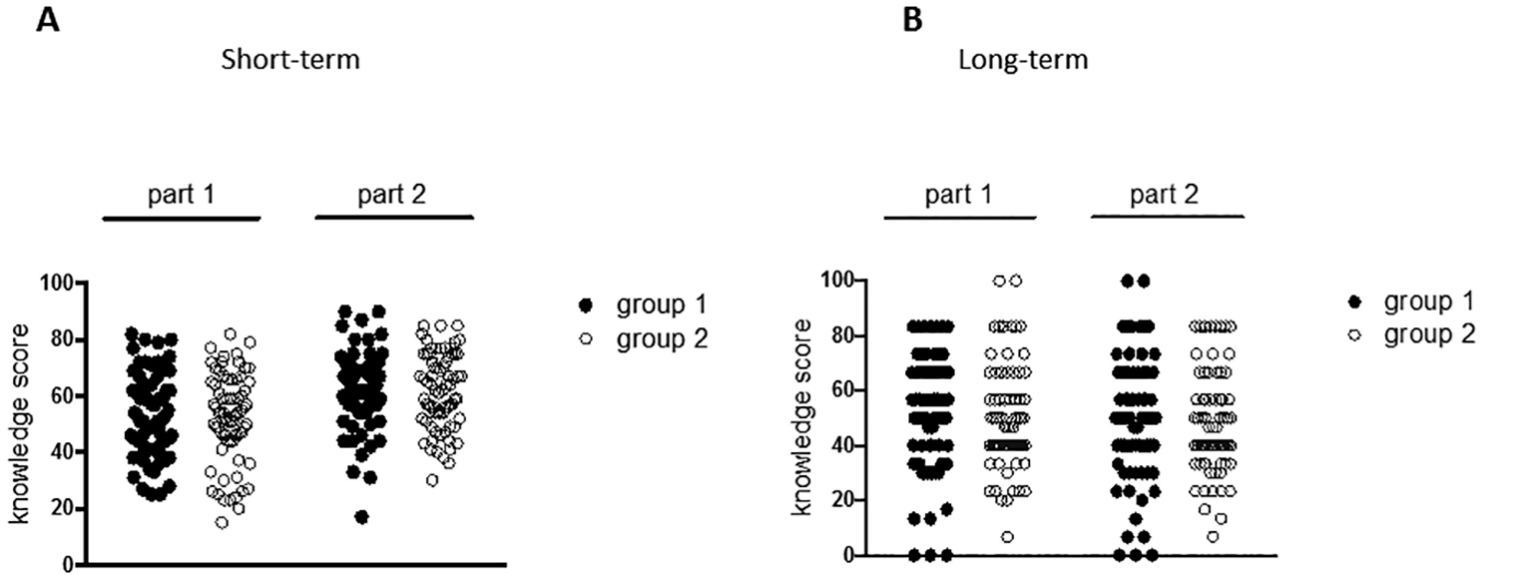
Evaluation of the effects of the ARS application on knowledge acquisition and retention

There was no significant difference in the short-term acquisition of knowledge in the first part of the program between groups (95% CI of the mean: group 1 [49.2; 56.2]; group 2 [48.6-55.9]; Mann-Withney p=ns). No difference was observed neither between the two groups in questionnaires evaluating the second part of the program (95% CI of the mean: group 1 [58.2-64.5]; group 2 [58.6-64.8]; Mann-Withney p=ns; Figure 3A). There was no significant difference between the two groups when only the six questions that were identical to those used during the lectures were considered.

There was no significant difference in the long-term retention of knowledge between the two groups in questionnaires assessing the first part of the program (group 1 95% CI of the mean: 45.7-55.4; group 2 95% CI of the mean: 45.8-54.6; Mann-Withney p=ns) and in questionnaires assessing the second part of the program (group 1 95% CI of the mean: 44.3-55.0; group 2 95% CI of the mean: 44.7-53.7; Mann-Withney p=ns; Figure 3B).

(A) Knowledge acquisition was evaluated using multiple choice questionnaires during the faculty exam (short-term evaluation) and (B) 3 months after the last lecture (long-term evaluation). The plots show the results of the students (on a 0 to 100 scale) according to group and to the part of the program evaluated.

## Discussion

This cross-over study shows that the use of an ARS smartphone application in medical education is feasible and improves the interactivity of the lectures, but that this does not translate to measurable improvement in knowledge acquisition or retention.

Compared to ARS using specific devices, a freely available ARS application engages no additional cost for the university, and does not require prior preparation of equipment. It does, however, require that the students have access to the technology. The proportion of medical students owning a smartphone is variable depending on the country, but will probably reach exhaustivity in the coming years. It is of note, however, that the smartphone penetration among medical students seems to be above that in the general population (86% in the present study vs. 70% in France ( *Multimédias − Tableaux de l’économie française | Insee*, no date)). Moreover, applications such as Socrative ^TM^ can be used on other devices such as tablets or laptops. Hence, the access to the application doesn’t seem to be a strong limitation of ARS applications at least in developed countries. Another potential limitation of the use of smartphone is the increase in battery usage which can penalize students who have not charged their phones before sessions ( Siddiqui, Iqbal and Azizi, 2017), but no student complained about this during this study. For the teacher, the use of the Socrative ^TM^ application is intuitive, and does not require additional time as compared to the preparation of an interactive lecture. Altogether, the use of an ARS application is feasible in medical education.

One potential caveat of using ARS application on smartphones is a decrease in student concentration during the lecture. The risk of distraction is not negligible with smartphones, as students mostly use them for entertainment and for socialization ( Lepp *et al.*, 2015). Accordingly, smartphone use has been associated with impaired performance among first year faculty students ( Baert *et al.*, 2018). However, most of the students herein agreed with the proposition that the use of the ARS application increases their level of concentration, although this is based on self-assessment, and not on objective measurement. A more objective assessment of the effects of ARS application use on student’s concentration could be interesting in future studies.

When assessing the first level of the Kirkpatrick model ( Kirkpatrick, 1977), i.e. the reaction of the students, the use of the ARS application seems highly useful. The large majority or the students included in this study were satisfied by the use of the ARS application, which is in line with a previous report ( Nelson *et al.*, 2012). Most of the students agreed that the use of an ARS application increases interactivity. As underlined in a recent meta-analysis, most of the previous studies reporting the positive effects of ARS on interactivity were biased because they compared interactive lecture with ARS versus non-interactive lecture. Herein, the lectures were exactly the same, with frequent solicitations of the audience with or without the ARS application. Hence, we can conclude that the ARS application by itself increases the interactivity of the lecture. Both teachers involved in this study (CL and PS) also reported that the use of the ARS application facilitates interactions within these relatively large classrooms (data not shown). Whereas this conclusion is valid for smaller or larger groups warrants further investigation.

Despite these benefits, there was no significant improvement in knowledge acquisition with the use of an ARS application. The absence of difference on short-term knowledge acquisition during the faculty exam may be explained by the relatively small effect of the ARS application as compared to other factors such as the intensity of personal work during revision. This bias does not, however, explain the absence of effect on long-term knowledge retention because the students were not aware of this evaluation. Another potential explanation for the lack of effect is that the positive effects of the ARS application might have been negated by the adverse effects of smartphone use. We can also evoke a methodological limitation of the knowledge evaluation used herein, as the ARS application might enhance reasoning capabilities of the students that are not easy to measure with multiple choice questionnaires.

## Conclusion

To conclude, this study demonstrates that the use of ARS smartphone application is feasible in medical education. Even if we were not able to detect any effect on knowledge evaluation, we encourage their use because it clearly increases the satisfaction of the students on the first level of the Kirkpatrick scale.

## Take Home Messages

• The use of an ARS application for smartphone is feasible in undergraduate medical lectures
• The use of an ARS application for smartphone increases the concentration of the students and the interactivity of the lecture
• The use of an ARS application for smartphone does not improve neither knowledge acquisition nor knowledge retention

## Notes On Contributors

Camille Lours (PharmD) is a junior teacher in hematology.

Pierre Sujobert (MD, PhD) is assistant professor in hematology. He coordinates the hematology module for undergraduates medical students in Lyon Sud faculty (Lyon university).
