# Supplementary material for: Crossover trial of an audience response system application for smartphone in undergraduate medical students
Source: MedEdPublish (2016). 2018 Sep 18;7:215. [Version 1] doi: 10.15694/mep.2018.0000215.1 (PMC10704420; doi:10.15694/mep.2018.0000215.1)
Supplement: Peer review history

# Reviewer report: Reviewer response for version 1

**DOI:** 10.21956/mep.19654.r29317

**Published:** 2019-07-01

**Authors:** Daniel Coyle

**Affiliations:** 1 NUI Galway

This review has been migrated. The reviewer awarded 4 stars out of 5 Mobile technologies are essential tools of medical education. As a medical student, I find that the use of interactive learning devices to be a welcome additive, to enrich concentration and collaboration among students. I have been privy to the use of clickers, lecture apps and online collaboration rooms, which are increasing in popularity every year.The topic is truly relevant to medical education as the Audience Response System (ARS) application is being used in an educational context. The application is supplementing the lecture material and it provides learning feedback to the students. The Abstract is very accurate and provides a vibrant description of the paper’s information. The statistical data reported is reflective of the objective set out in the paper. The layout of the trial is clearly outlined in the Methods section of the study.The Introduction supplies an abundant amount of background to the paper. It is well referenced to relevant literature, and to the specific objectives of the paper. In particular, the reference to the Bergtrom (2006) and Caldwell (2007) papers which provided evidence that the Audience Response System application increases the level of attention of the students. Although the Methods (along with the Outcome measurements) have been adequately described, a stepwise lay out would allow accurate trial replication. The Results are laid out in a forthright format. The authors follow a dedicated and logical outline including diagrams, tables and figures to display the resulting data. Each diagram or graph contained a brief description as a useful key and analysis of the information. It is understandable to the statistically minded as it contains difficult to interpret concepts such as 95% confidence intervals, p-values, Mann-Withney tests and ratios. More elaboration on the statistics would be beneficial to the lay reader. The Discussion is deep and intricate. The authors acknowledge that the strengths in their study lie mainly in the large satisfaction report of the students when using the ARS application. One could argue that pointing out the limitations in the methodology of the study would improve the overall paper. This an essential element of a research article. The Conclusion is quite short and sums up the overall information of the study simply. The authors do not reference the specific data in the results and discussion sections. Furthermore, the authors have identified an issue with the study, “The use of an ARS application for smartphone does not improve neither knowledge acquisition nor knowledge retention.” Regardless, they still recommend the use of ARS smartphone application because of the increase in student satisfaction levels. However, the authors have not provided a solution to the knowledge retention issue.There are no organizational issues, logical fallacies, or misconceptions of the referenced literature or resulting data. Additionally, the standard of English in this manuscript is quite good. I recommend that this article is a useful contribution to the medical education field that should be read by those with an interest in this area, particularly, medical professors, lecturers and tutors. Other target readers could include medical students who have a keen interest in medical education. If the authors decide to revise the paper, I would suggest that they should include the limitations of the study and outline the possible solutions or alternatives to the problems in the conclusion.

Reviewer Expertise:

NA

No decision status is available

# Reviewer report: Reviewer response for version 1

**DOI:** 10.21956/mep.19654.r29315

**Published:** 2019-01-23

**Authors:** P Ravi Shankar

**Affiliations:** 1 American International Medical University

This review has been migrated. The reviewer awarded 4 stars out of 5 I enjoyed reading this well-written manuscript. Audience-response technology (ARS) is being increasingly used in medical education. Traditionally it has involved the use of external devices (clickers). The recent introduction of Socrative (a software application) may enable more widespread use of ARS as there is no requirement for investing in specific technology. The study has been well designed and presented. The standard of written English is good. In a few places the use of language can be improved. The study will be of interest to all medical educators.

Reviewer Expertise:

NA

No decision status is available

# Reviewer report: Reviewer response for version 1

**DOI:** 10.21956/mep.19654.r29314

**Published:** 2019-01-22

**Authors:** Joaquín García-Estañ

**Affiliations:** 1 Universidad de Murcia

This review has been migrated. The reviewer awarded 4 stars out of 5 It is a very interesting study, albeit the analysis could have involved the rest of the topics in the course, just to see if the topic/professor has any effect on the outcomes. On the other hand, have you tried to use it for a whole academic year as a way to assess class attendance? Have you compared the app (Socrative) with Google Forms, for instance? What are the main differences?

Reviewer Expertise:

NA

No decision status is available

# Reviewer report: Reviewer response for version 1

**DOI:** 10.21956/mep.19654.r29316

**Published:** 2018-10-03

**Authors:** Thomas Puthiaparampil

**Affiliations:** 1 UNIVERSITI MALAYSIA SARAWAK

This review has been migrated. The reviewer awarded 3 stars out of 5 It is a highly relevant and interesting study in today's technology-driven world. Distraction during the teaching session by ARS is a potent drawback, but the possibility using it for interaction is encouraging. This study showed that the student performance was unaffected by the use of ARS. I consider the possible benefit of using the same facility for the students to ask questions and clarify doubts in a teaching sessions. This will be advantageous to improve the students' performance.

Reviewer Expertise:

NA

No decision status is available
